# Insights into extracellular vesicles: From biogenesis and tumor immune modulation to their clinical potential in immunotherapy

**DOI:** 10.1016/j.omtn.2026.103116

**Published:** 2026-09-26

**Authors:** Shuang Ma, Aoyan Qi, Jiaxuan Wu, Yue Niu, Wanying Xu, Xiaolin Wu, Yi Liu, Tao Yan

**Affiliations:** 1 School of Information Science and Engineering, Shenyang Ligong University, Shenyang 110159, China; 2 School of Mathematics and Statistics, Liaoning University, Shenyang 110036, China; 3 Department of Anesthesiology, Shanxi Province Cancer Hospital/Shanxi Hospital Affiliated to Cancer Hospital, Chinese Academy of Medical Sciences/Cancer Hospital Affiliated to Shanxi Medical University, Taiyuan, China; 4 Department of Anesthesiology, National Cancer Center/National Clinical Research Center for Cancer/Cancer Hospital, Chinese Academy of Medical Sciences and Peking Union Medical College, Beijing 100021, China

**Keywords:** MT: Bioinformatics, extracellular vesicles, EV-nucleic acids, liquid biopsy, RNA sorting, drug delivery systems, personalized immunotherapy

## Abstract

Extracellular vesicles (EVs) constitute a key mode of intercellular communication that remodels the complex tumor immune microenvironment by delivering diverse bioactive cargoes between tumor and immune cells. Nucleic acid species, encompassing microRNAs (miRNAs), messenger RNA (mRNAs), and other RNA transcripts, are functionally potent EV cargoes whose selective packaging dictates downstream immune signaling output. This review describes the molecular mechanisms driving selective RNA sorting into EVs, emphasizing sequence-motif recognition and core RNA-binding proteins FMR1 and TRIM25, which govern the enrichment of distinct RNA populations. We illustrate how EV-shuttled RNA molecules elicit context-dependent functional changes across regulatory T cells, myeloid-derived suppressor cells, and macrophages to shape antitumor or pro-tumor immune states. It critically integrates liquid biopsy evidence for EV-borne RNAs and EV-associated programmed death-ligand 1 (PD-L1) as predictive biomarkers of immunotherapy response in melanoma, non-small cell lung cancer, and hepatocellular carcinoma and dissects technical confounders such as non-vesicular RNA contamination and inconsistent isolation protocols. This review further compares nucleic acid loading strategies for native EVs versus cell-membrane-mimetic nanovesicles and explores their applicability for mRNA-based nanovaccines and combinatorial immunotherapy. We highlight underappreciated translational obstacles, noting that harmonized analytical standards, strict encapsulated cargo validation, and independent prospective multicenter trials are essential to translate EV-nucleic acid signatures into personalized cancer immunotherapy.

## Graphical abstract

**Figure:**
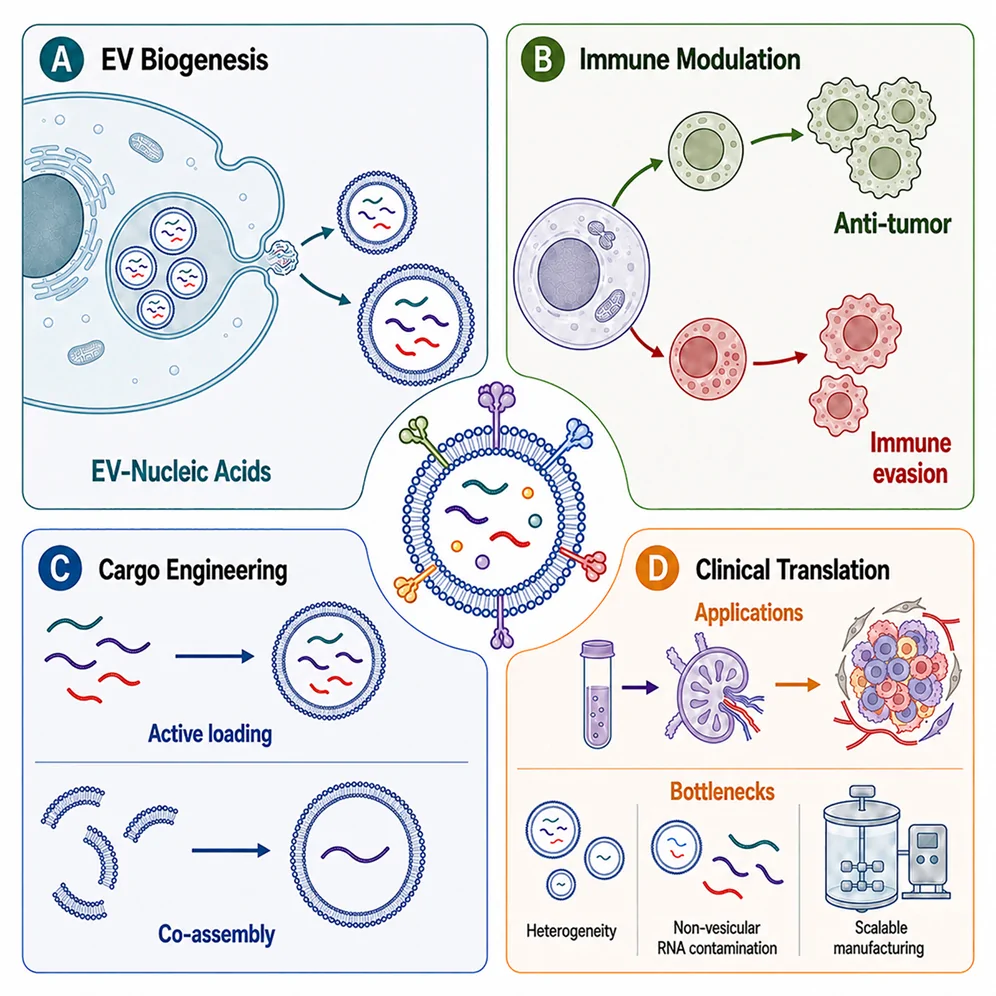

## Teaser

Yan and his colleagues review how RNA cargo packaged in extracellular vesicles remodels the tumor immune microenvironment. They discuss technical challenges limiting biomarker utility, compare nucleic acid loading strategies for engineered vesicles, and outline major translational obstacles hindering clinical adoption of EV-nucleic-acid-based personalized cancer immunotherapy.

## Introduction

Extracellular vesicles (EVs) are nanoscale membranous vesicles actively released by various cell lineages and are widely present in bodily fluids, including blood, urine, saliva, and cerebrospinal fluid. Based on their biogenesis mechanisms and particle size characteristics, EVs are heterogeneous and should be described primarily using operational terms, such as small EVs (sEVs; <200 nm) and large EVs (lEVs; >200 nm), unless their biogenetic origin has been directly demonstrated.^1^^,^^2^ EVs can encapsulate a variety of bioactive molecules, including proteins, lipids, messenger RNA (mRNA), microRNA (miRNA), and deoxyribonucleic acid fragments. As key mediators of intercellular information transmission, they play crucial roles in multiple pathological and physiological processes, such as immune regulation, tissue homeostasis maintenance, and disease progression.^3^ Over the past few years, the regulatory function of EVs in the tumor immune microenvironment has emerged as a research focus. Tumor-derived EVs can induce immune escape, promoting tumor progression and metastasis by presenting antigens, expressing immunosuppressive molecules, or regulating the functions of immune cells.^4^ EVs derived from immune cells can activate T cells and enhance antitumor immune responses, demonstrating their bidirectional regulatory role in tumor immunotherapy.^5^

With the continuous advancement of EV isolation and engineering technologies, their potential application as drug delivery carriers has become increasingly prominent. EV-mediated interactions do not necessarily exhibit universal intrinsic targeting but can be rendered more selective through engineered recognition systems. For example, EV-internalizing receptors expressed in dendritic cell progenitors enhanced the uptake of GD2-positive tumor-derived EVs and improved the efficacy of programmed cell death protein 1 (PD-1) blockade in mouse models.^6^ In addition, a variety of molecular markers carried on the surface or interior of EVs, such as programmed death-ligand 1 (PD-L1) and miRNA, have been confirmed to act as predictive biomarkers for immunotherapy responses, facilitating the development of personalized treatment strategies.^7^ The application of EVs in combination immunotherapy strategies has also shown promising prospects. Studies have demonstrated that EVs can synergize with immune checkpoint inhibitors (ICIs), photothermal/photodynamic therapy (PTT/PDT), or chemotherapy to remodel the immunosuppressive microenvironment, enhance antitumor immune responses, overcome the limitations of single-agent therapy, and improve therapeutic efficacy.^8^

This study reviews the biological basis, biosynthesis, and release mechanisms of EVs, as well as their roles in tumor immunity. This review focuses on the application prospects of EVs in tumor immunotherapy, including their function as drug carriers, clinical application as predictive biomarkers for immunotherapy, and utilization in immunotherapy-related combined cancer treatment strategies. Through an in-depth analysis of the multiple functions and potential applications of EVs, this review aims to provide a theoretical basis and practical guidance for the development of novel EV-based tumor immunotherapy strategies.

Substantial gaps remain in our understanding of the mechanisms, sorting regulation, and clinical translation of EV nucleic acids within the tumor immune microenvironment. Addressing these gaps and aligning with advances in nucleic acid therapeutics, this review makes three principal contributions. First, whereas most previous studies have focused on EV proteins, we center on EV-associated nucleic acids, including miRNAs, small interfering RNAs (siRNAs), mRNAs, and clustered regularly interspaced short palindromic repeat (CRISPR)-CRISPR-associated protein (Cas) systems. We systematically describe the mechanisms governing RNA sorting into EVs, move beyond the field’s predominant emphasis on PD-L1, and establish a nucleic acid-centered framework for EV-mediated intercellular communication. Second, we critically synthesize clinical-cohort evidence on EV nucleic acids as liquid biopsy biomarkers worldwide. By integrating studies with heterogeneous methods and conflicting findings, we highlight key technical barriers, including standardization of EV isolation, establishment of clinically meaningful cutoffs, and contamination by non-vesicular RNA. Finally, we distinguish natural EV-based drug loading from cell-membrane-mimetic vesicle systems and examine the therapeutic synergy of EV-delivered nucleic acids with immune checkpoint blockade, PTT, and PDT. This comparison clarifies the distinct applications of the two delivery strategies and provides a direction for optimizing next-generation personalized cancer immunotherapy.

## Biological basis of EV-nucleic acids

### Classification and characteristics of EVs

According to the MISEV2023 guidelines, the classification of EVs is shifting from traditional biogenesis-based nomenclature toward operational categories defined by physical properties. EVs should therefore be classified primarily by size as sEVs or lEVs. Although traditional EV subtypes, including exosomes, microvesicles, and apoptotic bodies, remain historically important, the limited specificity of current isolation methods often prevents experimental phenotypes from being linked confidently to a particular biogenetic pathway. Routine methods such as differential centrifugation and size-exclusion chromatography (SEC) separate particles primarily by size and density and cannot, by themselves, determine vesicle origin.^2^ Consequently, assigning origin-implying terms such as “exosomes” or “microvesicles” to vesicle populations obtained solely by physical separation remains scientifically contentious. This review therefore follows the MISEV2023 operational nomenclature and uses traditional subtype terms only when the original study directly verified the relevant biogenetic pathway. Current consensus also recognizes that commonly used markers—such as CD63 and TSG101 in sEV-enriched preparations, CD40L in some lEV populations, and phosphatidylserine exposed on multiple vesicle types—are not absolutely subtype specific. Their enrichment depends on both the isolation method and the physiological state of the source cells; no single marker should therefore be used as an absolute criterion for defining an EV subtype.^9^^,^^10^^,^^11^^,^^12^ Future characterization should integrate physical properties, molecular composition, and biogenetic evidence to classify vesicle populations accurately.

### Biosynthesis and release mechanisms of EVs

EV biogenesis is a highly complex intracellular process. For nucleic acid therapeutics, however, the decisive step is not vesicle formation itself but the active sorting and enrichment of nucleic acid cargo, particularly miRNAs, siRNAs, and mRNAs. Conventional physical classifications based on vesicle size provide limited insight into EV-mediated transfer of genetic information.^2^ Current research has therefore shifted toward determining how specific RNA subsets are selectively incorporated into nascent vesicles through highly specific molecular pathways. This active sorting establishes EVs as naturally programmable nucleic acid carriers and is governed mainly by three pathways.^13^^,^^14^ Selective RNA loading into EVs is regulated by sequence motifs and RNA-binding proteins. In particular, FMR1 and TRIM25 contribute to the selective enrichment of defined miRNA subsets in EVs.^15^^,^^16^

### The role of EV-nucleic acids in tumor immunity

As key mediators of intercellular communication, EVs play essential roles in tumor immunity, and many of these effects are mediated by the horizontal transfer of nucleic acid cargo. Although previous work has frequently emphasized surface proteins such as PD-L1, emerging evidence indicates that EV-encapsulated nucleic acids—particularly EV-associated miRNAs (EV-miRNAs)—are central regulators of tumor microenvironment (TME) reprogramming. By coordinately modulating signaling pathways within defined immune cell populations, EV-miRNAs can exert complex and context-dependent effects. This section describes EV biogenesis and operational classification together with their major roles in immune effector activation, EV-RNA-mediated regulation, and remodeling of the tumor immune microenvironment (Figure 1).

**Figure 1:**
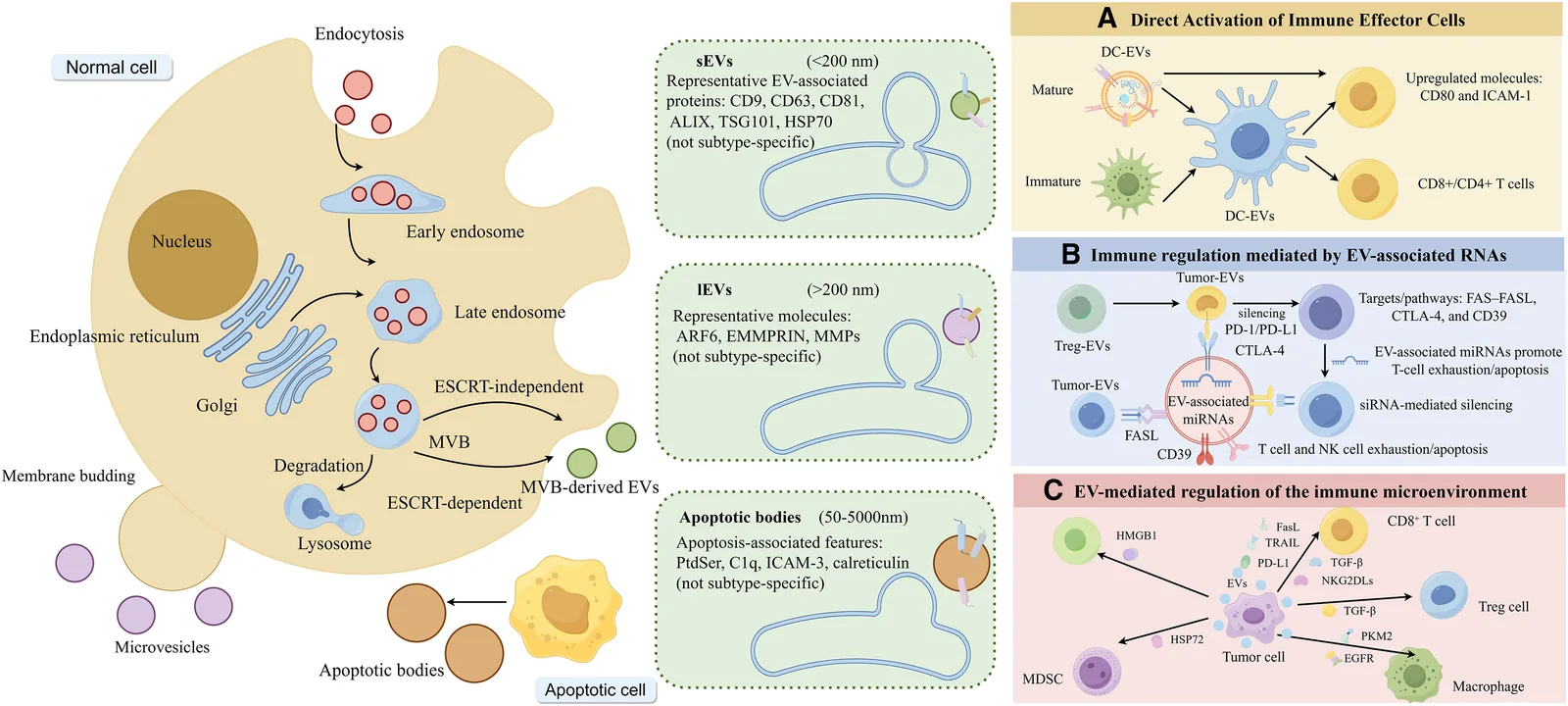
Biogenesis of extracellular vesicles and their regulatory roles in tumor immunity Exosomes are primarily generated through the endocytic pathway and the formation and release of multivesicular body (MVB), with RNA cargo sorted via endosomal sorting complex required for transport (ESCRT)-dependent and ESCRT-independent pathways. Microvesicles are formed by direct budding and detachment of the plasma membrane, whereas apoptotic bodies are produced during apoptosis. Representative markers associated with each EV subtype are also listed. (A) EVs directly activate immune effector cells. (B) EV-associated miRNAs mediate immune regulation. (C) EVs regulate the tumor immune microenvironment through their molecular cargo. This figure was created using Figdraw.

### Context-dependent dual immunoregulatory effects of EVs-RNAs

Within the tumor immune microenvironment, the tumor-promoting or tumor-suppressive effects of EVs are dynamic rather than fixed and are determined by multiple contextual variables.^4^^,^^5^^,^^17^ Their functional orientation depends on cellular lineage—for example, dendritic cell-derived EVs may promote activation, whereas tumor-cell-derived EVs may favor tolerance—and on cargo composition. In particular, the abundance of specific miRNAs and the level of PD-L1 influence signaling thresholds at the immune synapse. Microenvironmental stresses such as hypoxia also reshape EV release kinetics and cargo enrichment. Clinical interpretation must further distinguish tumor grade from clinical stage: the former reflects intrinsic secretory and biological properties, whereas the latter reflects systemic tumor burden. Thus, cell of origin, cargo composition, microenvironmental conditions, and disease characteristics collectively determine the context-dependent functions of EVs.^18^

### EV-RNAs induce the activation of immune effector cells

T cell activation has traditionally been considered dependent on direct receptor-mediated contact with antigen-presenting cells. Dendritic cell-derived EVs can transfer structurally intact peptide-loaded major histocompatibility complex molecules and other immunomodulatory cargo to recipient immune cells, thereby contributing to antigen presentation and T cell responses.^19^^,^^20^^,^^21^^,^^22^^,^^23^ In a distinct regulatory context, regulatory T cell (Treg)-derived EVs can transfer functional miRNAs that suppress pathogenic CD4+ T cell responses.^24^ Beyond T cells, EV nucleic acids can influence natural killer (NK) cells and macrophages through post-transcriptional regulation, promoting macrophage polarization toward an antitumor M1-like phenotype and enhancing NK cell recognition of tumor targets and release of cytotoxic granules.^17^ Such multidimensional, RNA-mediated activation of immune cell networks provides a molecular basis for durable systemic antitumor immunity (Figure 1A).

### Immune regulation mediated by EV-miRNAs and mRNAs

As key mediators of intercellular communication, EVs regulate immune responses through surface molecules and intraluminal bioactive cargoes, thereby shaping tumor immunity and immune homeostasis (Figure 1B).^17^

#### miRNA-dependent regulation

A major mechanism by which Treg-derived EVs exert suppressive activity is the direct delivery of functional miRNAs to recipient cells. Studies using genetically modified Treg cells have shown that EV-mediated miRNA transfer, rather than surface receptor signaling alone, is required for efficient suppression of pathogenic CD4+ T cell responses.^24^^,^^25^^,^^26^^,^^27^

#### Cytokine network reprogramming by EV RNAs

Tumor-derived EVs can horizontally transfer specific miRNAs that act at the post-transcriptional level in effector T cells. By suppressing pathways involved in interferon and interleukin production, these RNAs may induce a profound immunosuppressive state that cannot be explained solely by cytokine-receptor interactions.^28^^,^^29^

### EVs in the regulation of the immune microenvironment

The TME is a heterogeneous ecosystem in which tumor-derived EVs participate in local and systemic intercellular communication.^30^ Rather than acting exclusively through surface-receptor interactions, tumor-derived EVs serve as efficient RNA carriers that transfer selected miRNAs and other non-coding RNAs capable of reprogramming the local immune stroma and promoting immune escape (Figure 1C).^31^^,^^32^

### Regulation of immunosuppressive cells by EV-miRNAs

Tumor-derived EVs can drive the expansion and functional remodeling of local immunosuppressive cells through the transfer of immunoregulatory cargo. For example, tumor-derived exosomal high-mobility group box 1 can promote the expansion of TIM-1+ regulatory B cells and facilitate immune evasion in hepatocellular carcinoma (HCC).^33^ EV cargoes can remodel immunosuppressive myeloid cell populations through distinct molecular mechanisms. Tumor-derived EV-miRNAs can promote myeloid-derived suppressor cell (MDSC)-mediated immunosuppression and resistance to immunotherapy,^29^ whereas HCC-derived ectosomal PKM2 induces monocyte-to-macrophage differentiation and remodels the TME to support tumor progression.^34^ This remodeling capacity is closely related to the intrinsic biological properties of the tumor. Spatial transcriptomic profiles within the TME vary across tumor phenotypes and are associated with differences in disease behavior,^35^ while tumor-derived miRNA-containing EVs can induce phenotypic and transcriptional changes in recipient cells within the TME.^32^ Specific inhibitory RNAs may also recruit and expand MDSCs, thereby reinforcing immune tolerance within the TME.^29^

### The role of EV-RNAs in tumor immune evasion

Tumor-derived EVs are important drivers of immune escape. Although early studies emphasized EV-surface PD-L1, nucleic acid-centered models suggest that selectively packaged inhibitory RNAs also contribute substantially to disruption of the immune synapse.^18^ Tumor-associated EVs can promote immune evasion through several cargo- and context-dependent mechanisms. Circulating sEVs can exhibit adenosinergic activity that contributes to an immunosuppressive extracellular environment.^36^ sEVs isolated from the plasma of patients with triple-negative breast cancer can induce apoptosis of activated CD4+ and CD8+ T cells through EV-associated immunoregulatory surface molecules.^37^ Tumor-derived exosomes can also antagonize innate antiviral immune signaling in recipient cells.^38^ Collectively, these mechanisms position tumor-associated EVs as important mediators of impaired immune surveillance and acquired immune tolerance.

### Preparation of engineered EV carriers and drug loading

Extensive preclinical research has established EVs as promising cell-free delivery systems for cancer therapy.^39^^,^^40^ EVs possess favorable biocompatibility, low immunogenicity, and an ability to traverse certain tissue barriers; engineering can further enable efficient encapsulation and targeted delivery of macromolecular nucleic acids.^41^^,^^42^ Because unprotected RNA is rapidly degraded by circulating nucleases, the EV lipid bilayer provides a protective *in vivo* environment. Surface functionalization with targeting ligands can additionally promote selective homing to tumor or immune cells. The following sections describe the manufacturing and cargo-loading strategies used to convert natural EVs into programmable, targeted nucleic acid nanocarriers.

### Advantages of EVs as drug carriers

EVs have emerged as promising cell-free delivery systems for cancer therapy. Compared with synthetic nanocarriers such as liposomes, EVs offer several intrinsic advantages, including biological origin, favorable biocompatibility, low immunogenicity, and the ability to cross certain biological barriers.^39^^,^^40^^,^^41^^,^^42^^,^^43^ Natural EVs, however, do not possess universal tumor-specific targeting and are rapidly cleared from the circulation by the mononuclear phagocyte system in the liver, spleen, and lungs. Effective tumor targeting therefore usually requires surface peptide modification or genetic engineering, although limited tissue tropism has been observed for some cell-specific EV populations. Engineered EVs can facilitate cargo loading and targeted delivery, promoting intracellular release while protecting therapeutic agents from enzymatic degradation.^41^^,^^42^^,^^43^ EVs derived from stem cells and from allogeneic or autologous cells are being explored in preclinical and clinical studies.^41^^,^^42^ Their safety, stability, and delivery efficiency after engineering support the potential of EV-based nanodelivery strategies across an expanding range of cancer models.^42^^,^^43^

## Preparation of engineered EVs

### Isolation methods for preserving EV-nucleic acid integrity

#### Differential centrifugation

This method separates EVs through sequential centrifugation based on differences in particle size and density (Table 1). It is inexpensive, straightforward, compatible with large sample volumes, and remains one of the most common approaches for EV enrichment.^44^^,^^45^^,^^46^^,^^47^^,^^48^ However, it is time-consuming, and strong mechanical shear may disrupt vesicles and cause leakage of internal nucleic acids such as miRNAs and mRNAs. More importantly, differential centrifugation can co-pellet ribonucleoprotein (RNP) complexes, free RNA, and other contaminants, producing only low-to-moderate purity and compromising accurate quantification of EV-specific nucleic acid signals.^52^^,^^53^^,^^54^

**Table 1:** Comparison of common EV isolation and purification methods

Isolation approach	Isolation principle	Advantage	Disadvantage	Purity	Recovery	Reference
Differential centrifugation	sequential centrifugation according to particle size and density	low cost; simple operation; suitable for large sample volumes	time-consuming; co-pellets proteins, RNPs, free RNA, and other contaminants; possible shear-related vesicle damage	low	moderate	Nigro et al.,^44^ Zhou et al.,^45^ Langevin et al.,^46^ Oeyen et al.,^47^ Xu et al.^48^
Density gradient centrifugation	separation according to buoyant density in sucrose or iodixanol gradients	high purity; improved resolution of EV subpopulations; removes many proteins and lipoproteins	labor-intensive; low throughput; relatively low recovery; gradient material may require removal	high	low	Nigro et al.,^44^ Zhou et al.,^45^ Langevin et al.,^46^ Oeyen et al.,^47^ Xu et al.^48^
Size-exclusion chromatography (SEC)	separation according to particle size or molecular dimensions	preserves vesicle structure and biological activity; SEC removes many small soluble molecules	membrane clogging or possible vesicle damage during filtration; incomplete separation from similarly sized lipoproteins	high	moderate	Langevin et al.,^46^ Oeyen et al.,^47^ Xu et al.^48^
Polymer precipitation	PEG reduces EV solubility and promotes precipitation	rapid; simple; inexpensive; suitable for large-scale processing	co-precipitates proteins, lipids, polymers, and non-vesicular RNA; low purity and high analytical background	low	high	Sim et al.^49^
Immunoaffinity capture	specific binding between EV-surface antigens and antibodies or affinity ligands	high specificity, sensitivity, and purity; enables enrichment of defined EV subpopulations	relatively expensive; limited sample capacity; performance depends on antigen/ligand selection	high	low	Fitzgerald et al.,^50^ Wang et al.^51^

#### Density gradient centrifugation

EVs are separated according to their buoyant density in a gradient medium, enabling high-purity isolation and improved resolution of particle subpopulations (Table 1). This approach can remove many lipoproteins and protein complexes and thereby substantially improve sample purity.^44^^,^^45^^,^^46^^,^^47^^,^^48^ For nucleic acid studies, such purification is important for excluding non-vesicular RNA and for demonstrating that genetic cargo—including biomarker miRNAs or therapeutic constructs—resides within EVs.^52^^,^^53^^,^^54^ The method is nevertheless labor-intensive, low throughput, costly, and associated with relatively low recovery.

#### Size-based separation

Methods such as ultrafiltration and SEC separate EVs primarily by particle size or molecular dimensions (Table 1). They can enrich defined particle-size ranges while preserving vesicle integrity and biological activity.^46^^,^^47^^,^^48^ Because SEC avoids extreme physical stress, it generally preserves EV-associated nucleic acids and can remove many small soluble molecules from blood, making it useful in the preparation of engineered nucleic acid carriers. Limitations include membrane clogging in filtration-based workflows, possible vesicle damage, consumable costs, incomplete separation from similarly sized lipoproteins, and restricted scalability.^52^^,^^53^^,^^54^

#### Polymer precipitation

Polyethylene glycol (PEG) reduces solubility and promotes EV precipitation (Table 1).^49^ The method is rapid, simple, and cost-effective and does not require expensive equipment for large-scale processing. However, PEG also precipitates lipids, proteins, polymers, and non-membranous circulating RNAs, resulting in substantial contamination and low particle purity. This nonspecific enrichment creates high background noise in downstream nucleic acid sequencing and qualitative analyses and is therefore unsuitable for rigorous pharmacokinetic studies of EV-associated nucleic acids.

#### Immunoaffinity chromatography

This approach isolates vesicles through specific binding between EV-surface antigens and antibodies or other ligands (Table 1). It provides high specificity, sensitivity, and purity while preserving vesicle structure and biological activity.^50^ Such selectivity can be used to enrich EV subpopulations carrying defined functional nucleic acids, including therapeutic siRNAs or diagnostic miRNA signatures. The method is less suitable for large sample volumes and remains relatively expensive, although aptamer-based capture strategies may alleviate some of these limitations.^50^^,^^51^

### Active nucleic acid loading into EVs

Current delivery systems for therapeutic nucleic acids can be divided into two fundamentally distinct engineering platforms: cargo loading into naturally secreted EVs and the fabrication of cell-membrane-mimetic vesicles.^55^^,^^56^^,^^57^^,^^58^ Cargo can be introduced into natural EVs through passive or active loading. Passive loading by simple incubation is operationally convenient but inefficient for large, hydrophilic, or highly negatively charged molecules such as nucleic acids, due to the intrinsic properties of the EV membrane.^55^^,^^56^ Active loading, in which physical or chemical methods transiently disrupt the membrane to facilitate cargo entry before membrane resealing, has therefore become a principal strategy for natural-EV-based nucleic acid delivery.^58^ Common approaches tailored to RNA and DNA include sonication, electroporation, and device-assisted loading.

#### Sonication

Transient disruption of the EV membrane by ultrasound allows small RNAs to be encapsulated as the membrane reseals. This method is particularly effective for loading siRNAs and miRNA mimics and can support intracellular accumulation and subsequent target-gene silencing *in vivo*. However, it may induce EV aggregation and therefore requires additional purification.^56^

#### Electroporation

Short electrical pulses create temporary pores in the EV membrane, allowing highly negatively charged nucleic acids to enter the vesicle lumen. Electroporation is widely used to load siRNAs, miRNAs, and larger constructs such as CRISPR-Cas9 plasmids.^55^^,^^56^^,^^57^^,^^58^ Appropriately optimized electroporated EVs can protect nucleic acids from serum nucleases and facilitate intracellular delivery, although aggregation, cargo precipitation, and loading artifacts must be carefully controlled.

#### Device-assisted loading

Microfluidic and acoustofluidic devices provide precise, high-throughput loading. Inspired by lipid nanoparticle manufacturing, microfluidic mixing has been adapted to EVs to improve the encapsulation of macromolecular mRNAs and sensitive siRNAs. These platforms can enhance process uniformity and cellular uptake while maintaining membrane integrity, but their performance and scalability require further validation.^56^

## Cell membrane-derived EV mimetics

In contrast to post-isolation loading of natural vesicles, cell-membrane-mimetic vesicles are generated through an artificial self-assembly process.^55^^,^^56^^,^^57^ To address the low yield of natural EVs and the inefficient encapsulation of large, complex nucleic acids such as mRNA and CRISPR-Cas9 systems, this approach uses parental cell membranes and incorporates genetic material during vesicle reconstruction. Extrusion is currently a representative method for scalable production and high-capacity nucleic acid encapsulation.^58^

Extrusion: repeated filtration can assemble parental cell membranes into EV-like nanovesicles. For large and labile nucleic acids, including mRNA vaccines and CRISPR-Cas9 systems, extrusion allows genetic material to be co-assembled during vesicle formation. This strategy may overcome the limited yield of natural EVs and provide scalable, high-capacity encapsulation.^58^ Optimized formulations can preserve cargo integrity, reduce systemic clearance, and support effective *in vivo* translation of mRNA.

Each engineering method offers distinct advantages and limitations for nucleic acid delivery. In natural EVs, sonication and electroporation can substantially improve the loading of small nucleic acids such as siRNAs and miRNAs, but process parameters must be carefully optimized to preserve vesicle integrity and avoid aggregation or cargo precipitation. Device-assisted methods such as microfluidics offer uniform and efficient loading but require specialized and costly equipment. By contrast, extrusion-based assembly of biomimetic vesicles addresses the yield limitations of natural EVs and provides a potentially scalable, high-capacity option for large mRNAs and CRISPR plasmids, although absolute encapsulation efficiency and product consistency remain important challenges. Selection of an optimal strategy should therefore consider nucleic acid size and chemistry, the source of the carrier, manufacturing scalability, *in vivo* targeting, and the intended therapeutic effect.^55^^,^^56^^,^^57^^,^^58^

## Application of EV-nucleic acids in tumor immunotherapy

Owing to their multiple advantages as drug carriers, EVs exhibit considerable potential in drug delivery. This section summarizes their broad applications in tumor immunotherapy, including predictive biomarkers, nanovaccine platforms, and combination strategies (Figure 2).^59^ Specific biological and clinical claims below are supported by the corresponding original studies.^60^

**Figure 2:**
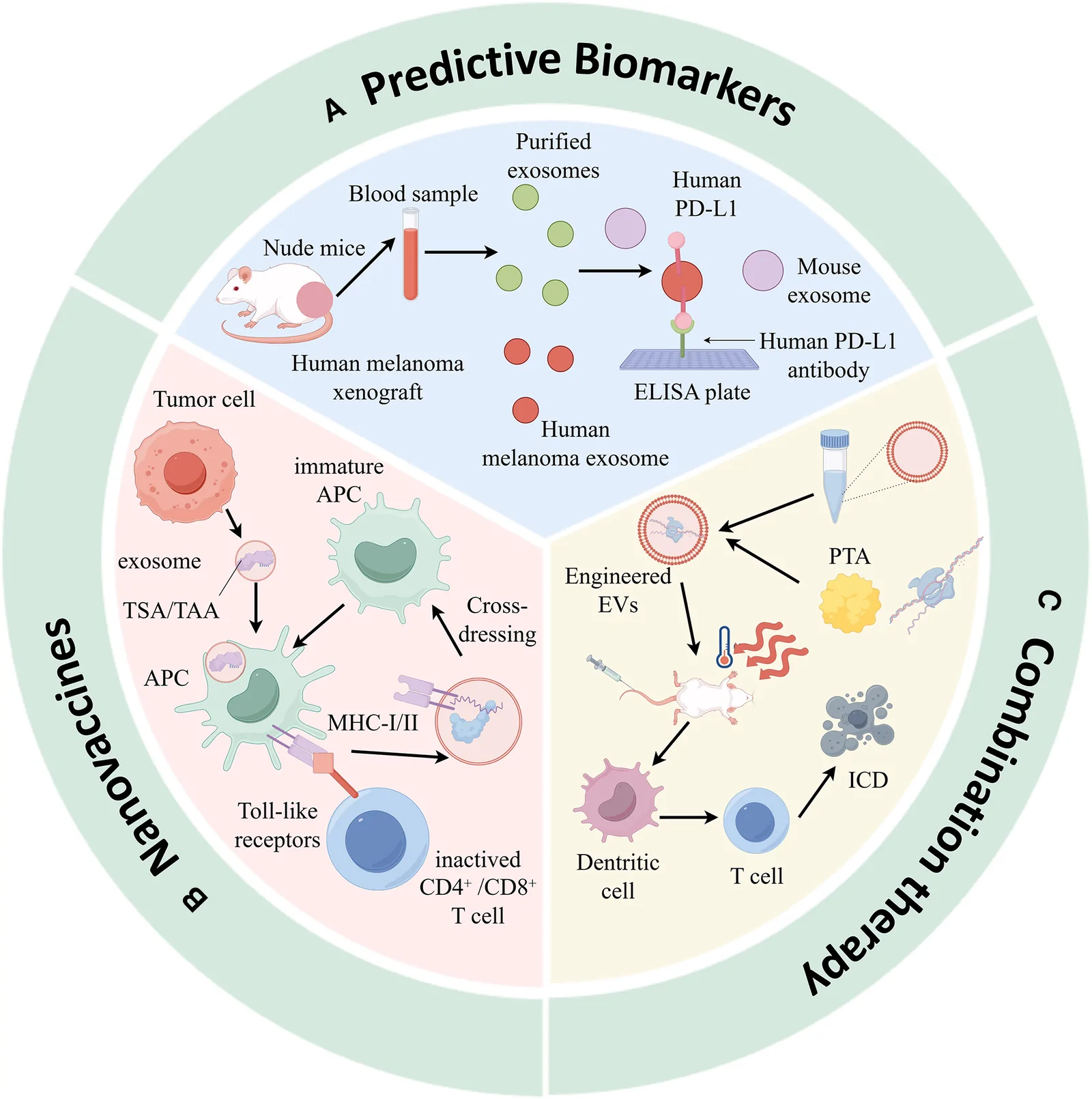
Application of EVs in tumor immunotherapy (A) PD-L1 is a predictive indicator of immunotherapy response. (B) Engineered dendritic cell-derived exosomes (EmDEX@GA) are used as nanovaccines in immunotherapy. (C) Engineered EVs have been used in combination immunotherapy with other therapies. This figure was generated by Figdraw.

### EV-miRNAs as predictive biomarkers for immunotherapy

EV-miRNAs have emerged as candidate biomarkers for predicting and monitoring responses to ICIs.^7^ They may integrate tumor-intrinsic signals with immune cell states while remaining detectable in serial liquid biopsies. Compared with a single surface protein such as PD-L1, multivariable miRNA signatures may capture several components of the cancer-immunity cycle.^29^^,^^61^ Their apparent performance, however, depends heavily on cohort composition, response definitions, pre-analytical sample handling, EV isolation, RNA extraction, normalization, and modeling strategy. The quantitative performance of each signature must therefore be interpreted within its original clinical and analytical context rather than generalized across tumor types. Representative EV protein and miRNA biomarkers are summarized in Table 2.

**Table 2:** Extracellular vesicles in melanoma and lung cancer as predictors of clinical response to immunotherapy

Tumor type	Key biomarker	EV source	Separation method	Prediction content	Key clinical findings	Reference
**EV protein biomarkers (PD-L1)**						

Melanoma	PD-L1	melanoma cell-derived EVs	differential centrifugation and exosome isolation kit	prediction of response to pembrolizumab	higher pretreatment exosomal PD-L1 was associated with nonresponse; an early ≥2.43-fold increase during pembrolizumab distinguished responders from nonresponders (80% sensitivity; 89% specificity)	Chen et al.^18^
Melanoma	PD1+EVs, PD-L1+EVs	melanoma cells, CD8+ T cells, B cells	ultracentrifugation with cell-source phenotyping	prediction of resistance and survival during anti-PD-1 therapy	higher melanoma cell/T cell PD-L1+ EVs and PD-1+ EVs were associated with nonresponse and adverse survival outcomes; selected cell-specific EV populations showed independent predictive value	Serratì et al.^61^
Melanoma	ΔPD-L1	plasma	differential centrifugation	monitoring treatment response and disease progression	longitudinal changes in exosomal PD-L1 were associated with progression-free and overall survival during melanoma treatment	Cordonnier et al.^62^
Melanoma	PD-1, CD28, CD80, CD86	T cells and dendritic cells	polymer-based exosome isolation with immunophenotyping	prediction of response to ipilimumab	higher baseline PD-1 and CD28 expression on T cell-derived exosomes was associated with clinical response and survival benefit during ipilimumab treatment	Tucci et al.^63^
NSCLC	PD-L1	plasma large EVs	differential centrifugation/large-EV enrichment	prediction of ICI response in tissue PD-L1-low or -negative disease	higher pretreatment PD-L1-positive large-EV levels were associated with improved immunotherapy response and survival, particularly when tissue PD-L1 was low or negative	Schöne et al.^64^
NSCLC	PD-L1	serum exosomes	MagCapture Exosome Isolation Kit PS	association with tumor immunity and anti-PD-1 response	serum exosomal PD-L1 correlated with CD8+ tumor-infiltrating lymphocytes and tissue PD-L1; the study evaluated its association with postoperative anti-PD-1 disease control and recurrence-free survival	Shimada et al.^65^
NSCLC	EV PD-L1 dynamics	plasma EVs	ultracentrifugation	prediction of durable clinical benefit during ICI therapy	on-treatment decreases in EV PD-L1 were associated with durable benefit, whereas increases were more frequent in nonresponders; EV PD-L1 dynamics outperformed tissue PD-L1 in the reported cohorts	de Miguel-Perez et al.^66^
EV nucleic acid biomarkers (miRNA)						
NSCLC	miR-199a-3p, miR-21-5p, miR-28-5p (plasma); miR-320b/c/d and miR-125b-5p (EV-enriched fraction)	plasma and EV-containing plasma fractions	size-exclusion chromatography	prediction of durable benefit from anti-PD-1/PD-L1 therapy	a three-miRNA plasma signature distinguished durable benefit from nonresponse (AUC 0.925); separate EV-enriched miRNA signals were associated with progressive disease or favorable response	Shukuya et al.^67^

Cross-study comparisons reveal substantial heterogeneity. Some studies classify stable disease as clinical benefit, whereas others restrict response to complete or partial response; cutoffs may be based on fold change, absolute abundance, or longitudinal ratios. Small discovery cohorts, incomplete external validation, and technical differences between RNA sequencing and targeted RT-qPCR may further inflate apparent performance. Methodological comparisons also indicate that SEC can provide relatively high EV recovery but does not completely remove lipoproteins, density gradient centrifugation improves purity at the expense of yield, and combined procedures may alter both the number and identity of detected miRNAs.^52^^,^^53^^,^^54^

#### Melanoma prediction

In melanoma, EV-surface PD-L1 and EV-miRNAs represent biologically and analytically distinct biomarker classes and should be evaluated separately (Figure 2A).^63^ In addition to longitudinal changes in EV-PD-L1 during melanoma treatment,^62^ a group of miRNAs associated with MDSCs—including miR-146a, miR-155, miR-125b, miR-100, let-7e, miR-125a, miR-146b, and miR-99b—may reflect immunosuppressive states and resistance to ICI therapy. A baseline circulating miRNA score comprising let-7e, miR-125a, miR-99b, miR-146b, and miR-125b was associated with poorer survival in patients treated with ipilimumab or nivolumab, whereas the same association was not observed in patients receiving targeted therapy. This finding suggests that the signature may reflect ICI-related immune tolerance rather than general disease prognosis.^29^

Overlap among reported melanoma miRNA candidates remains limited. Likely explanations include small cohorts, retrospective designs, mixed anti-CTLA-4 and anti-PD-1 regimens, inconsistent response endpoints and sampling schedules, and differences in tumor burden or immune cell composition. Moreover, some studies analyzed total plasma RNA or only partially enriched EV-containing fractions rather than rigorously purified and source-validated EVs. The predictive signal therefore cannot yet be assigned confidently to tumor EVs, immune cell EVs, or non-vesicular circulating RNA. Most studies also did not use orthogonal isolation methods to exclude lipoproteins and Argonaute-containing RNP complexes, which can alter apparent miRNA abundance and introduce substantial confounding.^52^^,^^53^^,^^54^

Before clinical sensitivity and specificity are reported, future studies should prespecify response endpoints, sampling times, and cutoffs; use reproducible EV-enrichment workflows; and quantify ApoA1, ApoB, and Argonaute contamination. Candidate miRNA models should lock variables, parameters, and decision thresholds in a discovery cohort and then be tested in an independent prospective cohort receiving a consistent treatment regimen. Negative findings should be reported alongside area under the receiver operating characteristic curve (AUC), sensitivity, and specificity with confidence intervals. Only under these conditions can the incremental predictive value of a miRNA panel beyond clinical variables and EV-PD-L1 be established.

#### Lung cancer prediction

In non-small cell lung cancer (NSCLC), Shukuya et al. evaluated pretreatment circulating miRNAs and miRNAs in EV-containing fractions from patients receiving anti-PD-1/PD-L1 therapy. A three-miRNA plasma signature characterized by lower miR-199a-3p, miR-21-5p, and miR-28-5p distinguished patients with durable clinical benefit from nonresponders, with an AUC of 0.925, compared with 0.575 for tissue PD-L1 expression.^67^ In the EV-containing fraction, miR-320b, miR-320c, and miR-320d were enriched in patients with progressive disease, whereas a treatment-associated decrease in the T cell-suppressive miR-125b-5p correlated with a favorable response.^67^ The study did not report locked-parameter clinical sensitivity and specificity for these EV-miRNA signals, and the sequencing cohort was small. Thus, the AUC of 0.925 should be attributed to the three-miRNA circulating plasma signature and not extrapolated to the miR-320/miR-125b-5p EV panel.

The NSCLC findings illustrate the reproducibility challenges in miRNA biomarker research. Candidate panels vary with histological subtype, line of therapy, response definition, sampling time, and whether total plasma or an EV-enriched fraction is analyzed.^67^ Models selected and evaluated in the same small discovery dataset also tend to overestimate AUC. Reliable clinical translation therefore requires locked multivariable panels, prespecified cutoffs, multicenter external validation, and simultaneous reporting of AUC, sensitivity, specificity, positive predictive value, and negative predictive value with confidence intervals.

Analytical purity is particularly important for abundant circulating miRNAs such as miR-21 and miR-122 because high- and low-density lipoproteins (HDLs/LDLs) and Argonaute-containing RNP complexes may co-isolate with EVs. Polymer precipitation often maximizes RNA yield but provides low particle purity, ultracentrifugation may induce protein aggregation and does not completely separate lipoproteins, and SEC performance varies with pore size and fraction selection. These methodological differences alter both the amount of miRNA recovered and the apparent composition of a biomarker signature.

#### HCC prediction

In HCC, quantitative evidence for EV-miRNAs is currently stronger for diagnosis and prognosis than for predicting response to ICIs. Wang et al. reported that an enriched serum-EV panel comprising miR-122, miR-21, and miR-96 distinguished HCC from cirrhosis with an AUC of 0.924, 82% sensitivity, and 92% specificity, outperforming individual miRNAs and alpha-fetoprotein (AFP).^68^ This finding supports the diagnostic potential of HCC-associated EV-miRNA panels but must not be interpreted as evidence for predicting responses to anti-PD-1/PD-L1 therapy or antiangiogenic-immunotherapy combinations. Among the peer-reviewed clinical studies identified in this review, no HCC-specific EV-miRNA panel has yet been validated for ICI response while reporting AUC, sensitivity, and specificity together.

The absence of HCC-specific performance data for ICI response represents an important translational gap. Etiology—including hepatitis B virus, hepatitis C virus, alcohol-related disease, and metabolic liver disease—together with cirrhosis severity, hepatic inflammation, and the selected systemic regimen may alter circulating miRNA profiles independently of tumor response. Future studies should therefore develop regimen-specific signatures in prospectively collected cohorts and compare them with AFP, radiographic response, and established clinical variables. Discovery and validation sets should be independent, and both positive and negative findings should be reported to reduce publication bias.

The HCC diagnostic panel also highlights a source-specificity problem. miR-122 is highly abundant in the liver and is carried by both EVs and non-vesicular particles; altered levels may therefore reflect hepatocyte injury, cirrhosis, or lipoprotein-associated RNA rather than tumor-EV cargo.^68^ Before a predictive signal is attributed to membrane-enclosed EVs, orthogonal separation, RNase/protease protection assays, and ApoA1/ApoB and Argonaute controls are required.

Complementing the published biomarker studies summarized above, ongoing registered clinical investigations are evaluating EV-based liquid biopsy strategies across multiple tumor types. These include circulating-exosome analysis in melanoma (ClinicalTrials.gov: NCT05744076), plasma exosome or exosomal PD-L1 studies in NSCLC (ClinicalTrials.gov: NCT04427475 and NCT02890849), serum exosomal miRNA for predicting the efficacy and prognosis of immunotherapy combined with chemotherapy in lung squamous carcinoma (ClinicalTrials.gov: NCT05854030), circulating exosomes as companion diagnostic biomarkers in renal cell carcinoma (ClinicalTrials.gov: NCT05705583), and combined circulating tumor cell and exosome analysis for predicting immunotherapy efficacy in HCC (ClinicalTrials.gov: NCT05575622) (Table 3). Together, these studies demonstrate the breadth of current clinical interest in EV-based response prediction while also highlighting the need for standardized EV isolation, biomarker assays, response definitions, and prospective validation.

**Table 3:** Clinical trials of EVs as biomarkers for immunotherapy efficacy

NCT number	Trail title	Cancer type	Study type	Observational/interventional model	Ages eligible for study	Study start	Sponsor
NCT05744076	Analysis of Circulating Exosomes in Melanoma Patients	melanoma	observational	cohort	18 years and older (adult, older adult)	March 1, 2019	Center Hospitalier Universitaire de Besancon
NCT04427475	Study on the Prediction of Immunotherapeutic Effect of Advanced Non-small Cell Lung Cancer by Detection of Plasma Exosomes	NSCLC	interventional	parallel assignment	18 years and older (adult, older adult)	June 8, 2020	Fudan University
NCT02890849	Clinical Research for the Consistency Analysis of PD-L1 in Cancer Tissue and Plasma Exosome	NSCLC	interventional	single group assignment	(child, adult, older Adult)	October 1, 2016	Xinqiao Hospital of Chongqing
NCT05854030	A Study on Serum Exosomal miRNA Predicting the Effective and Prognosis of Immunotherapy Combined With Chemotherapy in Pulmonary Squamous Carcinoma	lung squamous carcinoma	observational	cohort	18 years and older (adult, older adult)	April 1, 2022	Tianjin Medical University Cancer Institute and Hospital
NCT05705583	A Companion Diagnostic Study to Develop Circulating Exosomes as Predictive Biomarkers for the Response to Immunotherapy in Renal Cell Carcinoma	renal cell carcinoma	observational	cohort	18 years and older (adult, older adult)	January 1, 2023	Zhejiang Cancer Hospital
NCT05575622	Clinical Study for Combined Analysis of CTC and Exosomes on Predicting the Efficacy of Immunotherapy in Patients With HCC	HCC	observational	cohort	18–80 years (adult, older adult)	January 1, 2023	Zhongnan Hospital

This table includes only clinical studies of ICI-related immune biomarkers.

## Critical perspectives: Contradictory clinical findings and methodological confounders

Despite considerable interest in EV-based biomarkers, the cancer immunotherapy literature is vulnerable to publication bias favoring positive results over negative or statistically nonsignificant findings. Prospective and retrospective studies have reported heterogeneous associations between EV-PD-L1 or EV-miRNA measurements and treatment response or survival in patients receiving immune checkpoint therapy.^64^^,^^65^^,^^66^^,^^67^^,^^68^ Several factors may explain these discrepancies. Differences in study design, including selection bias in retrospective cohorts versus stricter prospective protocols, and small sample sizes reduce statistical power. Inconsistent diagnostic cutoffs and heterogeneous analytical platforms—such as enzyme-linked immunosorbent assay versus single-molecule array for proteins and differing RT-qPCR or RNA sequencing workflows for nucleic acids—further impair reproducibility.^52^^,^^53^^,^^54^ More fundamentally, EV-isolation protocols often introduce substantial non-vesicular RNA contamination. Polymer precipitation and ultracentrifugation can co-isolate HDL, LDL, and RNP complexes, meaning that reported “EV-miRNA” signatures may represent heterogeneous mixtures of vesicular and freely circulating miRNAs. Such contamination dilutes genuine vesicular signals, introduces unpredictable confounding, and impedes meaningful cross-study comparison. Resolving these methodological confounders is a prerequisite for establishing the clinical utility of EV-encapsulated nucleic acids.

### Engineered EVs as mRNA nanovaccines for cancer immunotherapy

Conventional cancer vaccines often show limited clinical efficacy due to poor lymph node targeting and inefficient lysosomal escape. Engineered EVs may overcome these barriers and serve as systemic carriers for nucleic acid antigens, particularly mRNA vaccines. Extrusion or microfluidic loading can be used to generate dendritic cell-derived EV formulations containing mRNAs that encode tumor-associated antigens. Relative to conventional lipid nanoparticles, such EV-mRNA nanovaccines may offer structural stability and improved delivery to the tumor-draining lymph node, supporting antigen expression and sustained cytotoxic T lymphocyte (CTL) activation.

### Application of dendritic cell-derived exosomes in nanovaccines

Mature dendritic cell-derived exosomes (DEXs) naturally contain MHC class I/II molecules, co-stimulatory molecules, and adjuvant-like components, thereby transferring tumor antigens to recipient cells and promoting systemic immune activation (Figure 2B). However, single-agent DEXs have elicited limited immune responses in patients with cancer, supporting their combination with additional adjuvants to enhance CTL activity.^69^ Engineered DEXs such as EmDEX@GA incorporate C-C chemokine receptor type 7-dependent lymph node targeting, PD-1/PD-L1 blockade, and stimulator of interferon genes pathway activation to remodel tumor-draining lymph nodes and inhibit distant metastasis (Figure 3A).^70^ The IDNV-CPG (CpG-loaded induced pluripotent stem cell–dendritic cell hybrid nanovesicles) nanovaccine combines induced pluripotent stem cell membranes, DEXs, and a CpG adjuvant to target lymph nodes and dendritic cells, activate antitumor immunity, and inhibit tumor growth and metastasis (Figure 3B).^71^ Surface-engineered NK cell-derived EVs may likewise improve targeting and stability. Proposed strategies include coupling EVs with CTLs for TME-responsive cargo release to enhance local cytotoxicity and combining EVs with miRNA-loaded dendrimers for targeted therapy (Figure 3D).

**Figure 3:**
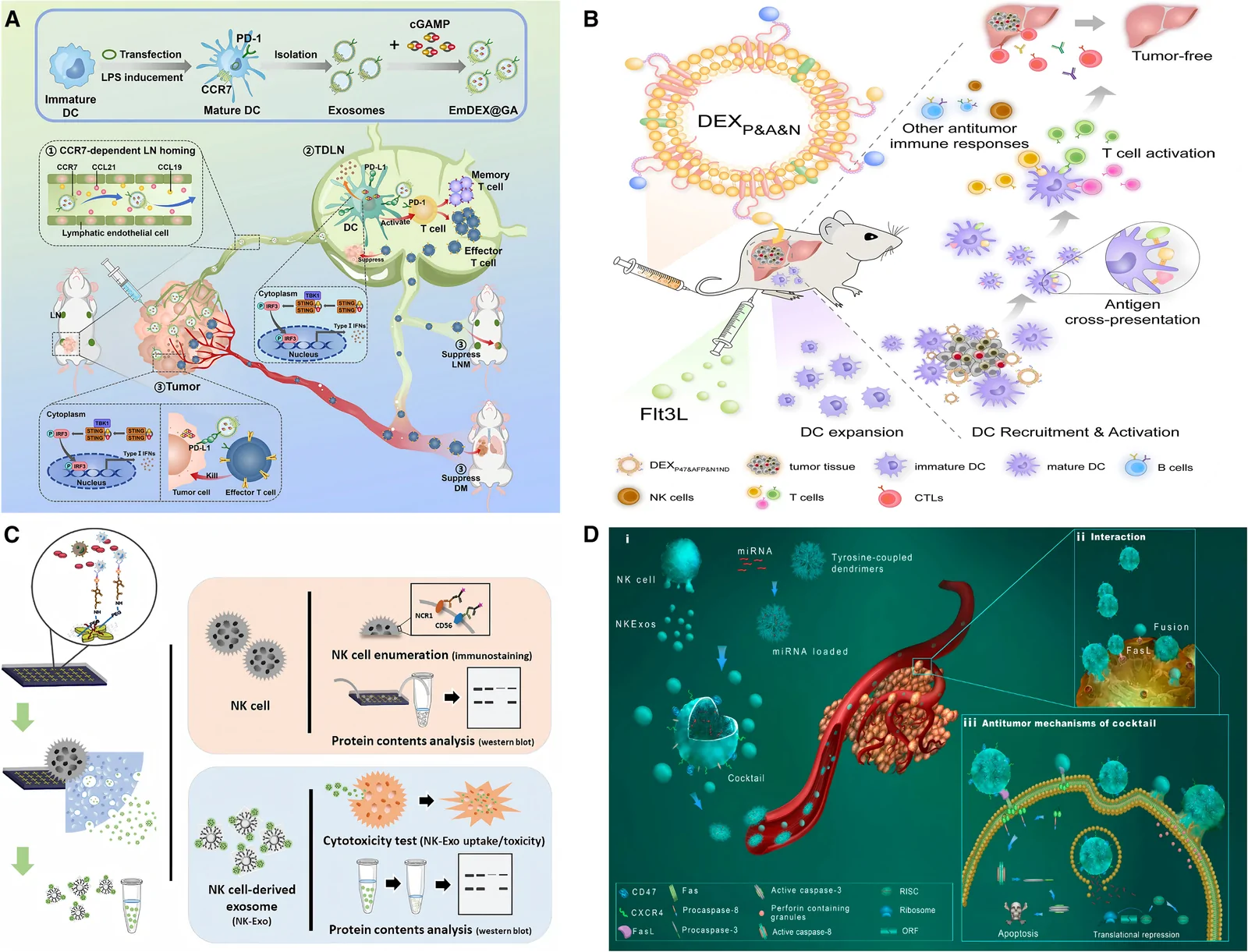
Engineered EVs as nanovaccines for cancer immunotherapy (A) Mechanism of EmDEX@GA in remodeling the local regional immune microenvironment to inhibit tumor progression and metastasis. (B) Synergistic hybrid induced pluripotent stem cell (iPSC)-dendritic cell (DC) vesicles for targeted activation of antitumor immunity. (C) NK cell-derived exosomes exhibit direct antitumoral activity against circulating tumor cells via cytotoxic proteins. (D) Exosome-camouflaged core–shell nanoparticles/natural killer cell-derived exosomes (NN/NKEXO) cocktail therapy for tumor targeting and drug delivery. This figure was post-processed and assembled using Adobe Illustrator 2023.

### Application of NK cell-derived exosomes in nanovaccines

NK cell-derived EVs contain cytotoxic molecules, including perforin, granzymes, and FasL, and can kill cancer cells. EVs released by expanded NK cells show cytotoxicity against ovarian cancer cells with limited toxicity toward normal cells (Figure 3C).^72^ Surface engineering can improve the targeting and stability of NK cell-derived EVs. Strategies under investigation include combining EVs with CTLs to enable TME-targeted cargo release and enhance local cytotoxicity, as well as integrating EVs with miRNA-loaded dendrimers for targeted therapy (Figure 3D).^73^ These findings support the potential of engineered NK cell-derived EVs as cancer immunotherapy platforms.

### Application of EV-nucleic acids in combined immunotherapy

Engineered EVs can co-deliver therapeutic nucleic acids and chemotherapeutic agents, thereby increasing tumor cell immunogenicity while reversing an immunosuppressive TME. A representative example is the dual-delivery Galectin-9 siRNA-loaded exosomes (iEXO)-oxaliplatin (OXA) system, which uses bone marrow mesenchymal stromal cell-derived EVs. Galectin-9 (Gal-9)-targeting siRNA is loaded by electroporation, and an OXA prodrug is conjugated to the vesicle surface (Figure 4A).^74^ OXA induces tumor cell death and can trigger immunogenic cell death (ICD), promoting calreticulin exposure and the release of damage-associated molecules such as high-mobility group box 1 and adenosine triphosphate. These signals enhance antigen uptake and dendritic cell maturation and subsequently promote effector-T cell recruitment and activation.

**Figure 4:**
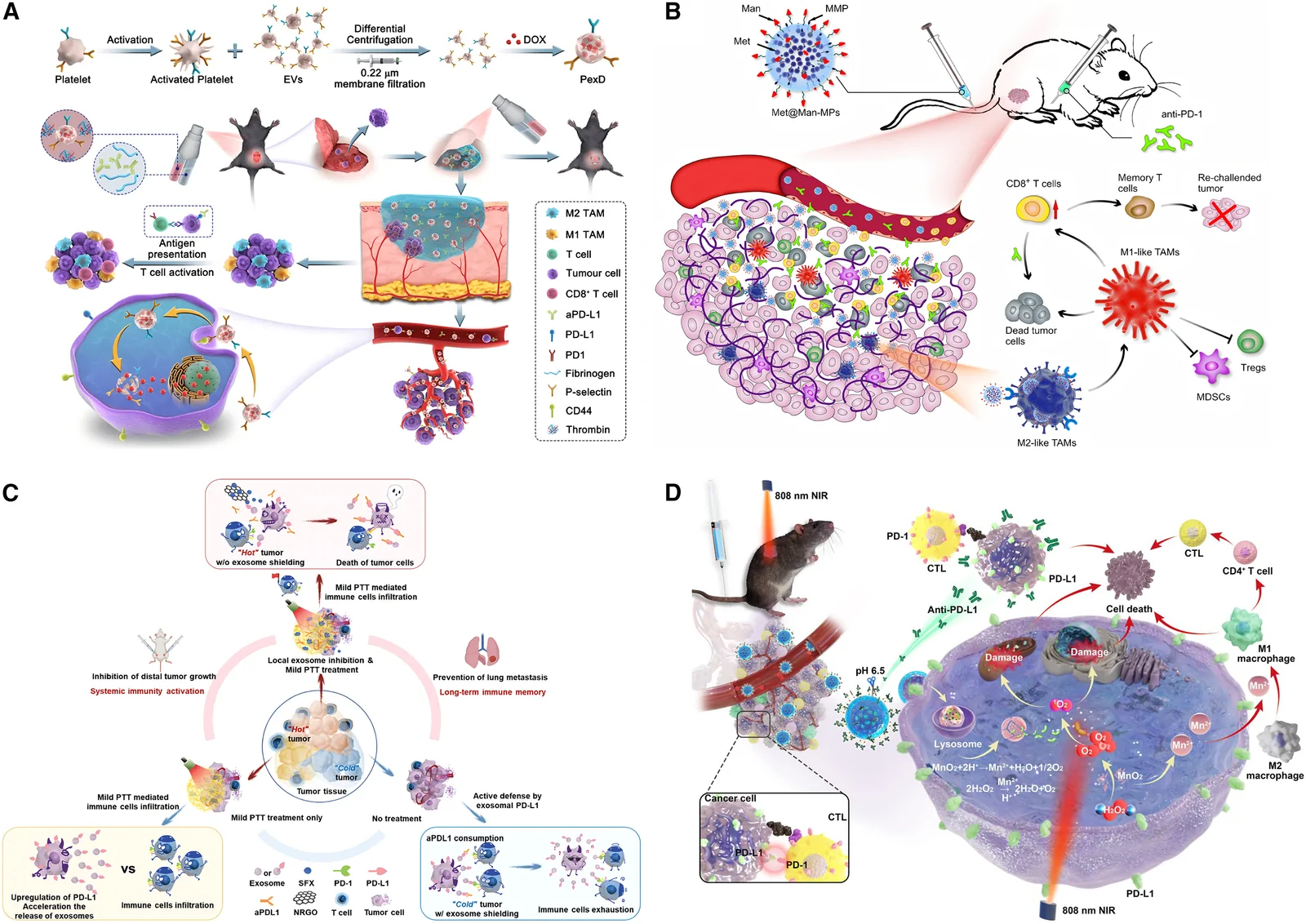
Application of EVs in immunotherapy combined with other therapeutic modalities (A) EVs encapsulating doxorubicin (DOX) and anti-PD-L1 monoclonal antibodies to induce ICD and inhibit local tumor recurrence and tumor metastasis. (B) Met@Man-MPs loaded with metformin to enhance anti-PD-1 therapy. (C) Combined immunotherapy based on mild PTT for remodeling the TME and exosome inhibition to enhance immune checkpoint blockade therapy. (D) ICG@MnO2@Exo-anti-PD-L1 combining PDT and immunotherapy for the treatment of NSCLC. This figure was adjusted and refined using Adobe Illustrator 2023.

Concurrently, EV-delivered Gal-9 siRNA reduces Gal-9 expression in tumor cells at the post-transcriptional level, disrupts the immunosuppressive Gal-9/Dectin-1 axis, and promotes repolarization of tumor-associated macrophages from an M2-like to an M1-like phenotype. The iEXO-OXA platform therefore integrates chemotherapy-induced ICD with nucleic acid-mediated remodeling of the immune microenvironment, creating a coordinated sequence of tumor-antigen release, antigen presentation, and T cell activation. In preclinical pancreatic cancer models, the strategy increased intratumoral drug accumulation and enhanced antitumor immunity, illustrating how EVs can coordinate chemotherapy with immunomodulation.^75^ These results remain predominantly preclinical, and loading consistency, biodistribution, safety, and scalable manufacturing require further evaluation.

### Combined immunotherapy and immune checkpoint blockade

ICI resistance is frequently associated with poor T cell infiltration and an immunosuppressive TME.^76^ Mannose-modified macrophage-derived microparticles loaded with metformin (Met@Man-MPs) targeted M2-like macrophages, promoted M1-like repolarization, increased CD8+ T cell infiltration, and enhanced anti-PD-1 efficacy with durable immune memory (Figure 4B).^77^

### Combined immunotherapy, PTT, and PDT

PDT generates reactive oxygen species (ROS)-mediated cytotoxicity, whereas PTT uses localized hyperthermia. In the cited engineered vesicle studies, these modalities were combined with immune checkpoint blockade to improve tumor-localized treatment and antitumor immune responses (Figure 2C).^78^^,^^79^ One multistage delivery system combined a photosensitizer, an anti-PD-L1 antibody, and an inhibitor of EV release. This strategy suppressed primary tumors, activated systemic immunity, inhibited distant tumor growth, prevented lung metastasis, and generated durable immune memory (Figure 4C).^78^ An indocyanine green (ICG)-containing platform (ICG@MnO2@Exo-anti-PD-L1) co-delivered the photosensitizer and an anti-PD-L1 antibody, improving tumor targeting and PDT efficacy, alleviating hypoxia, promoting M2-to-M1 macrophage repolarization, and suppressing tumor growth and metastasis with favorable biocompatibility (Figure 4D).^79^ Together, these findings support the synergistic integration of PDT/PTT with immunotherapy.^78^^,^^79^

## Conclusion

As key mediators of intercellular communication, EVs exert complex bidirectional effects on tumor immunity. Depending on their cellular origin, cargo composition, and the specific context of the TME, EVs may promote tumor growth, angiogenesis, and immune evasion or activate antitumor immune responses. This functional duality creates both opportunities and challenges for EV-based tumor immunotherapy. EVs have considerable potential as therapeutic carriers and predictive biomarkers. Their ability to transport chemotherapeutic agents, small-molecule inhibitors, miRNAs, and other therapeutic cargo enables targeted delivery, potentially improving treatment efficacy while reducing adverse effects. Engineered EVs may also enhance combination strategies involving chemotherapy, ICIs, PDT, and PTT. Meanwhile, EV-associated nucleic acids and surface proteins can reflect tumor-immune remodeling and may therefore help predict responses to immunotherapy. However, clinical translation remains limited by EV heterogeneity and methodological constraints. EVs from different sources vary substantially in size, composition, and function, complicating their standardized isolation, characterization, and evaluation. Additionally, these methods may compromise the structural integrity and biological function of isolated EVs. More efficient and reproducible purification technologies are therefore needed to preserve EV integrity and biological activity. Further optimization is also required to improve cargo-loading efficiency, targeting specificity, *in vivo* stability, and circulation duration. Attention should also be paid to the immunogenicity of EVs; although they are generally regarded as having low immunogenicity, they may still elicit immune responses under certain conditions, thereby compromising their therapeutic efficacy. Accordingly, the immune properties and safety of EVs must be systematically evaluated before clinical application. Despite this promise, the clinical translation of EVs remains constrained by major methodological challenges. In addition to EV heterogeneity, inefficient cargo loading, and potential immunogenicity,^80^^,^^81^ a central concern is contamination by non-vesicular RNA. The lack of standardized isolation and purification procedures allows free circulating miRNAs, lipoproteins, and RNP complexes to co-enrich with EV preparations. This systematic confounding can obscure genuine vesicle-mediated signals, hinder comparisons across independent cohorts, and contribute to repeated failures in the clinical validation of predictive biomarkers.^82^ To overcome these limitations, future biomarker and mechanistic studies should adhere strictly to international guidelines such as MISEV2023 and incorporate orthogonal separation methods—for example, SEC combined with density gradient centrifugation or immunoaffinity capture—to distinguish vesicle-enclosed cargo from non-membranous particles. Together with rigorous clinical validation, improved loading and targeting strategies, and standardized quality control, these advances will be essential for translating EV-based therapeutics and biomarkers into reliable clinical applications.

## Acknowledgments

This work was supported by National High Level Hospital Clinical Research Funding and National Cancer Center Climbing Fund (grant no. NCC202404005), the CAMS Innovation Fund for Medical Sciences (grant no. 2025-I2M-C&T-B-047), the National Natural Science Foundation of China (grant nos. 82674270), the National Key R&D Program of China (grant no. 2023YFC3403804), the National Science and Technology Major Project (grant nos. 2024ZD0533303 and 2024ZD0533300), the Bethune Charitable Foundation (kqxs-2024-A-02), and National High Level Hospital Clinical Research Funding (80102022619). The authors thank Home for Researchers (www.home-for-researchers.com).

## Author contributions

S.M. and A.Q. jointly conceived and designed the study and supervised the project. Y.N. drafted the manuscript and developed the methodology. W.X. and Y.L. conducted the literature review, interpreted the data, and contributed to manuscript translation. T.Y. and J.W. contributed to the development of the conceptual framework and data interpretation. X.W. contributed to the conceptual framework and performed critical revision and final editing of the manuscript. All authors read and approved the final manuscript and agree to be accountable for their respective contributions and for the integrity of the work.

## Declaration of interests

The authors declare no competing interests.

## Declaration of generative AI and AI-assisted technologies in the manuscript preparation process

During revision of this manuscript, an AI-assisted language tool was used to translate selected Chinese passages into English and to improve grammar and academic expression. The tool was not used to generate figures, process images, analyze data, or determine the scientific conclusions of the review. The authors reviewed and edited all translated content and take full responsibility for the accuracy, integrity, and final wording of the manuscript.
